# Supplementary material for: From sequence to enzyme mechanism using multi-label machine learning
Source: BMC Bioinformatics. 2014 May 19;15:150. doi: 10.1186/1471-2105-15-150 (PMC4229970; doi:10.1186/1471-2105-15-150)
Supplement: Additional file 2 — Java code of ml2db. Additional file ml2db_code.tar.gz contains the Java source code to run the multi-label machine learning experiments and save the results to database. The code’s Javadoc is included. [file 1471-2105-15-150-S2.zip › additional file 2/ml2db/ml2db/doc/mulan/classifier/meta/class-use/HierarchyBuilder.Method.html]

Uses of Class mulan.classifier.meta.HierarchyBuilder.Method


JavaScript is disabled on your browser.


- Overview
- Package
- Class
- Use
- Tree
- Deprecated
- Index
- Help

- Prev
- Next

- Frames
- No Frames

- All Classes

## Uses of Class mulan.classifier.meta.HierarchyBuilder.Method

- Packages that use HierarchyBuilder.Method

  | Package | Description |
  | --- | --- |
  |  |  |
  | --- | --- |
  | mulan.classifier.meta |  |
- - ### Uses of HierarchyBuilder.Method in mulan.classifier.meta

    Methods in mulan.classifier.meta that return HierarchyBuilder.Method

    | Modifier and Type | Method and Description |
    | --- | --- |
    |  |  |
    | --- | --- |
    | `static HierarchyBuilder.Method` | HierarchyBuilder.Method.`valueOf(java.lang.String name)` Returns the enum constant of this type with the specified name. |
    | `static HierarchyBuilder.Method[]` | HierarchyBuilder.Method.`values()` Returns an array containing the constants of this enum type, in the order they are declared. |

    Constructors in mulan.classifier.meta with parameters of type HierarchyBuilder.Method

    Constructor and Description

    `HierarchyBuilder(int partitions,
    HierarchyBuilder.Method method)`

    Constructs a new istance based on given number of partitions and method

    `HOMER(MultiLabelLearner mll,
    int clusters,
    HierarchyBuilder.Method method)`

    Creates a new instance based on given multi-label learner, number of
    children and partitioning method

- Overview
- Package
- Class
- Use
- Tree
- Deprecated
- Index
- Help

- Prev
- Next

- Frames
- No Frames

- All Classes
